# Development and validation of an interpretable machine learning model for early prediction of mortality in ICU patients with chronic obstructive pulmonary disease and heart failure

**DOI:** 10.1097/MD.0000000000051224

**Published:** 2026-10-23

**Authors:** Jiayi Shen, Yucheng Liu, Yikun Chen

**Affiliations:** a Department of Respiratory and Critical Care Medicine, Affiliated Hospital of Jiangsu University, Zhenjiang, Jiangsu, China.

**Keywords:** chronic obstructive pulmonary disease, heart failure, machine learning, MIMIC-IV database, mortality, predictive model

## Abstract

Chronic obstructive pulmonary disease and heart failure frequently coexist, and the mortality risk for patients with these comorbidities is significantly elevated in the intensive care unit (ICU) setting. Therefore, developing an interpretable model for early mortality prediction is crucial to improving the prognosis and management of these patients. This study developed models based on the Medical Information Mart for Intensive Care IV database (3167 patients) and performed external validation using the eICU Collaborative Research Database (1781 patients). Least absolute shrinkage and selection operator regression combined with the Boruta algorithm was used to screen clinical features within the first 24 hours of ICU admission. Based on the selected features, 5 machine learning models (eXtreme Gradient Boosting, Categorical Boosting, Light Gradient Boosting Machine, Random Forest, and Support Vector Machine) were constructed and compared for their performance in predicting 28-day all-cause mortality. The optimal model was interpreted using the Shapley Additive Explanations framework. In internal validation, the Categorical Boosting model demonstrated the best overall performance, achieving an area under the receiver operating characteristic curve of 0.788, with a sensitivity of 0.482 and a specificity of 0.900. In independent external validation, the model’s area under the receiver operating characteristic curve was 0.739. Feature selection identified 14 key predictive variables. Shapley Additive Explanations analysis revealed that the Sequential Organ Failure Assessment score, Simplified Acute Physiology Score II score, anion gap, vasopressor use, and respiratory rate were the top 5 features contributing most to the model’s predictions. This study developed and validated an interpretable machine learning model based on multicenter data for the early prediction of 28-day mortality risk in ICU patients with chronic obstructive pulmonary disease and heart failure. The model demonstrates good discriminative ability and reasonable generalizability, with its interpretability analysis clarifying core predictive factors to aid in clinical risk stratification and intervention.

## 1. Introduction

Chronic obstructive pulmonary disease (COPD) is a common chronic condition characterized by persistent respiratory symptoms and airflow limitation. It has a high global prevalence, affecting approximately 12% of the world’s population, and is the third leading cause of death worldwide, with about 3 million annual deaths.^[1,2]^ Concurrently, heart failure (HF) is a complex clinical syndrome resulting from impaired cardiac pump function, leading to inadequate tissue perfusion. It affects approximately 64 million people globally, posing a major public health challenge.^[3,4]^ COPD and HF frequently coexist, with epidemiological studies indicating that up to 30% to 40% of COPD patients also have HF.^[5]^ These conditions exacerbate each other pathophysiologically: chronic hypoxia and systemic inflammation in COPD increase cardiac load, whereas pulmonary congestion in HF further compromises lung function. Patients frequently present with overlapping symptoms, such as shortness of breath, which complicates diagnosis, treatment, and clinical management.^[6]^ In the intensive care unit (ICU) environment, patients with combined COPD and HF often present with more severe conditions and face higher short-term mortality risk.^[7]^ Therefore, early identification of high-risk patients is crucial for improving clinical decision-making and prognosis.

In recent years, machine learning, leveraging its ability to handle complex medical data, has become a key technology for enhancing disease prediction and clinical decision quality.^[8]^ In critical care, such techniques aid in the early identification of high-risk patients and optimization of treatment plans. However, many existing machine learning models lack interpretability, with internal decision processes difficult to comprehend, leading clinicians to be cautious about prediction results.^[9]^ Consequently, explainable artificial intelligence methods such as SHAP (Shapley Additive Explanations) have been proposed. These methods improve model interpretability by quantifying feature contributions, thereby facilitating the application of predictive results in clinical practice.^[10]^ Currently, while machine learning has shown potential in assessing outcomes for patients with COPD or HF individually, research specifically targeting the clinically high-risk population with COPD and HF comorbidity remains relatively scarce.^[11,12]^

Therefore, this study aims to develop and validate a clinical prediction model based on multicenter data from the Medical Information Mart for Intensive Care IV (MIMIC-IV) and eICU Collaborative Research Database (eICU-CRD). Using the LASSO-Boruta algorithm for feature selection and combining various machine learning methods, the model seeks to predict the 28-day mortality risk early in ICU patients with combined COPD and HF. Furthermore, the study will provide an interpretability analysis of the model’s decisions through the SHAP framework to assist in clinical risk stratification and intervention.

## 2. Methods

### 2.1. Data sources and study population

This study used data from the MIMIC-IV database (v3.1) and the eICU Collaborative Research Database (eICU-CRD, v2.0). MIMIC-IV is a large, publicly available ICU clinical database developed and maintained by the MIT Laboratory for Computational Physiology. It contains detailed, de-identified clinical data from critically ill patients admitted to Beth Israel Deaconess Medical Center in Boston, USA.^[13]^ The eICU-CRD is a multicenter critical care collaborative research database containing clinical data from ICUs across multiple hospitals in the United States. One of the authors (Jiayi Shen) has obtained access authorization for these 2 databases (ID: 68967720). As all patient information was de-identified, the study received an exemption from ethical review. COPD and HF were identified based on the International Classification of Diseases, Ninth and Tenth Revisions, diagnosis codes. Exclusion criteria included age <18 years, hospital stay <24 hours, non-first ICU admission record, and missing data exceeding 30% for key variables. Finally, 3167 patients from MIMIC-IV were included for analysis, and 1781 patients from eICU-CRD were included for external validation (Fig. 1).

**Figure 1.**
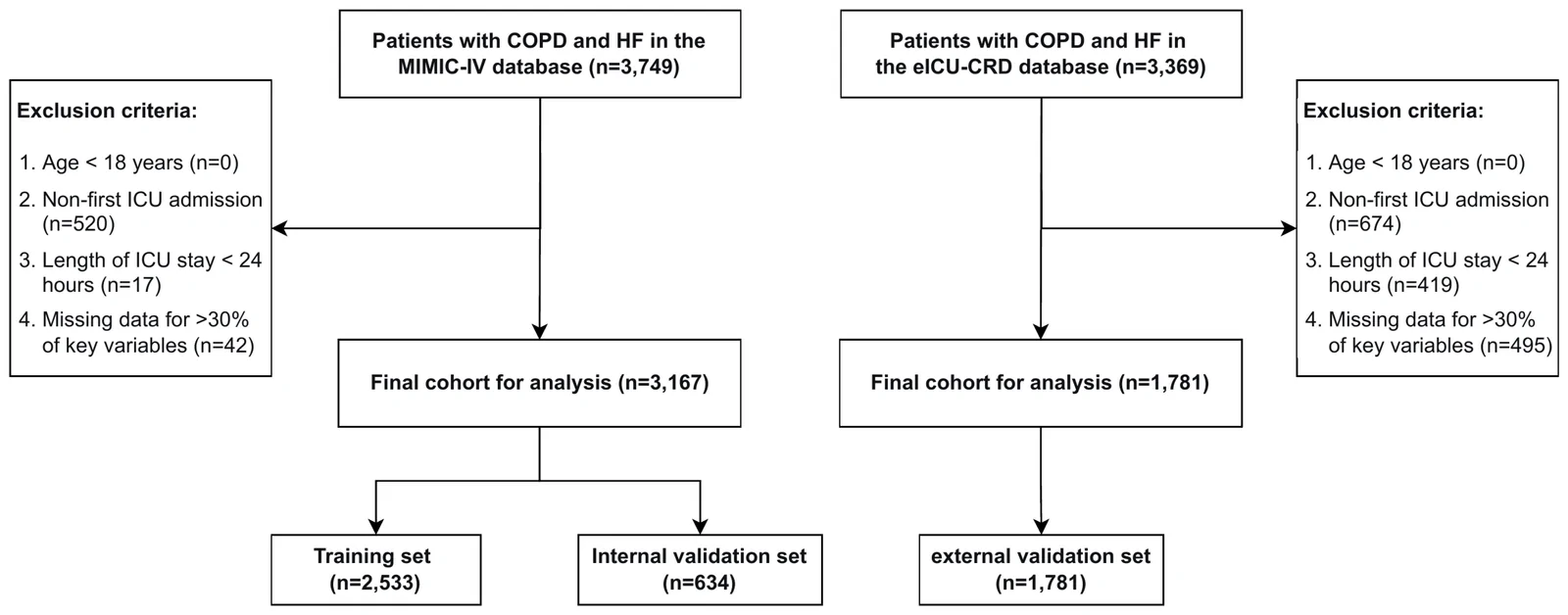
Flowchart of study population selection. COPD = chronic obstructive pulmonary disease, eICU-CRD = eICU Collaborative Research Database, HF = heart failure, ICU = intensive care unit, MIMIC-IV = Medical Information Mart for Intensive Care IV.

### 2.2. Data extraction and processing

Structured Query Language was used to extract clinical data for target patients from the MIMIC-IV and eICU-CRD databases. Extracted variables included demographic data (age, sex), disease severity scores (Sequential Organ Failure Assessment [SOFA], Simplified Acute Physiology Score II [SAPS II], Glasgow Coma Scale [GCS]), vital signs (heart rate [HR], systolic blood pressure, diastolic blood pressure, respiratory rate [RR], oxygen saturation, temperature), laboratory indicators (hemoglobin, platelets, red cell distribution width, red blood cell count, white blood cell count, anion gap [AG], calcium, chloride, glucose, potassium, sodium, international normalized ratio [INR], partial thromboplastin time, creatinine, blood urea nitrogen), comorbidities (hypertension, acute kidney injury, cirrhosis, cerebrovascular accident, chronic kidney disease, cancer, hyperlipidemia, myocardial infarction, ischemic heart disease), and treatment measures (mechanical ventilation, vasopressors, antibiotics, corticosteroids). All continuous variables were the average values within the first 24 hours after ICU admission. Variables with >20% missing values were excluded; the remainder were imputed using the K-nearest neighbors algorithm. To assess robustness, we performed sensitivity analyses using mean imputation and complete-case analysis (excluding patients with any missing values).

### 2.3. Study outcome

The study outcome was 28-day all-cause mortality for COPD patients complicated by HF following ICU admission. This outcome refers to the proportion of such patients who died from any cause within 28 days of the first ICU admission.

### 2.4. Data processing and feature selection

All data analysis in this study was performed on the DecisionLinnc1.0 platform (Statsape Co. Ltd.). DecisionLinnc1.0 is a platform integrating multiple programming language environments, enabling data processing, analysis, and machine learning through a visual interface.^[14]^ For baseline characteristic analysis, the normality of continuous variables was tested using the Kolmogorov–Smirnov test. Normally distributed data were described as mean ± standard deviation (μ ± σ), with group comparisons using the *t* test; non-normally distributed data were described as median (interquartile range) (M [Q1, Q3]), with group comparisons using the Mann–Whitney *U* test. Categorical data were described as numbers (percentages) (n [%]), and comparisons were performed using the χ^2^ test. Subsequently, the MIMIC-IV dataset was randomly split into a training set and an internal validation set in an 8:2 ratio. In the training set, least absolute shrinkage and selection operator (LASSO) regression was used for feature selection based on the criterion of minimum cross-validation error (λ.min). Simultaneously, the Boruta algorithm was used to assess the importance of all features by comparing them with randomly generated “shadow features” to identify key variables. Finally, the intersection of the results from the 2 screening methods was taken as the final set of predictive features.

### 2.5. Model development and comparison

To address the class imbalance in the outcome event data, the Synthetic Minority Over-sampling Technique was first applied to the training set. Subsequently, based on the selected features, 5 different machine learning models were constructed on the training set to predict the 28-day mortality risk in ICU patients with COPD complicated by HF. The models used included eXtreme Gradient Boosting, Categorical Boosting (CatBoost), Light Gradient Boosting Machine, Random Forest, and Support Vector Machine (SVM). All models were trained on the same training set using the default parameters provided by each algorithm library, and their performance was evaluated on the internal validation set. Model performance was comprehensively compared using the following metrics: area under the receiver operating characteristic curve (AUROC), F1 score, sensitivity, specificity, and accuracy. Additionally, decision curve analysis (DCA) and calibration curves were plotted to evaluate the clinical net benefit at different decision thresholds and the consistency between predicted probabilities and actual risk, respectively. Through a comprehensive comparison of these metrics, the model with optimal performance was selected for subsequent analysis.

### 2.6. External validation and model interpretability analysis

The best-performing model from the MIMIC-IV dataset was subjected to external validation on the independent eICU-CRD dataset to confirm its robustness in a new patient population. Furthermore, to enhance model understandability, the SHAP framework was employed for the interpretability analysis of the optimal model. This method quantifies the contribution of each predictor to the model outcome and presents key features and their directional impact visually, thereby providing an intuitive basis for clinical decision-making.

## 3. Results

### 3.1. Baseline characteristics of the study population

This study ultimately included 3167 ICU patients with COPD and HF from the MIMIC-IV database. Among them, 2608 patients survived and 559 were non-survivors, resulting in a 28-day mortality rate of 17.6%. As shown in Table 1, non-survivors were significantly older. Disease severity scores, including SOFA and SAPS II, were also significantly higher among non-survivors. Regarding vital signs, non-survivors had faster heart rates and RRs, while systolic and diastolic blood pressures were lower. Laboratory findings indicated that white blood cell count, AG, glucose, potassium, INR, partial thromboplastin time, creatinine, and blood urea nitrogen levels were all significantly higher in non-survivors, whereas hemoglobin, red blood cell count, and calcium levels were significantly lower. Collectively, these indicators suggest that non-survivors had more severe inflammation, anemia, electrolyte disturbances, coagulation dysfunction, and renal impairment. Regarding comorbidities, the proportions of acute kidney injury, chronic kidney disease, cirrhosis, myocardial infarction, and sepsis were significantly higher in non-survivors than in survivors. Regarding treatment measures, a higher proportion of non-survivors received vasopressors and antibiotics.

**Table 1:** Baseline characteristics of patients by 28-day mortality.

Variable	Levels	Overall (N = 3167)	Survivor (N = 2608)	Non-survivor (N = 559)	*P* value
General information					
Age (yr)		75 (67–82)	74 (67–82)	79 (71–86)	<.001
Gender, n (%)	Female	1477 (46.64)	1223 (46.89)	254 (45.44)	.531
	Male	1690 (53.36)	1385 (53.11)	305 (54.56)	
Vital signs					
HR (bpm)		83.21 (73.31–95.16)	82.22 (72.90–93.32)	88.90 (76.80–101.67)	<.001
SBP (mm Hg)		113.00 (104.04–124.00)	113.64 (104.95–124.90)	109.19 (101.00–119.37)	<.001
DBP (mm Hg)		61.46 (55.28–67.96)	61.73 (55.50–68.01)	60.39 (54.33–67.28)	.007
RR (breaths/min)		19.65 (17.44–22.15)	19.44 (17.30–21.85)	20.96 (18.13–23.88)	<.001
SPO_2_ (%)		96.27 (94.64–97.67)	96.27 (94.68–97.68)	96.31 (94.42–97.67)	.024
Temperature (°C)		36.77 (36.58–36.96)	36.77 (36.58–36.96)	36.75 (36.54–36.97)	.889
Score system					
SOFA		5 (3–7)	4 (3–7)	7 (5–10)	<.001
SAPS II		39 (32–48)	37 (31–45)	49 (40–59)	<.001
GCS		15 (14–15)	15 (14–15)	15 (13–15)	<.001
Laboratory results					
Hb (g/dL)		10.03 (8.75–11.50)	10.10 (8.78–11.60)	9.80 (8.58–11.15)	<.001
PLT (×10^9^/L)		193.00 (145.33–253.50)	194.42 (147.50–253.50)	186.33 (133.10–254.00)	.170
RDW (%)		15.50 (14.35–17.20)	15.40 (14.30–17.10)	16.07 (14.90–17.60)	<.001
RBC (×10^12^/L)		3.44 (2.99–3.99)	3.46 (3.02–4.02)	3.38 (2.91–3.84)	<.001
WBC (×10^9^/L)		10.90 (8.00–14.90)	10.67 (7.85–14.53)	12.25 (8.65–17.17)	<.001
AG (mmol/L)		14.00 (12.00–16.50)	13.71 (11.67–16.00)	15.40 (13.00–18.50)	<.001
Calcium (mg/dL)		8.50 (8.00–8.90)	8.50 (8.07–8.90)	8.33 (7.83–8.80)	<.001
Chloride (mmol/L)		101.67 (97.33–105.50)	102.00 (97.50–105.67)	101.00 (96.75–105.00)	.106
Glucose (mg/dL)		132.00 (109.25–167.00)	130.50 (109.00–164.50)	140.00 (113.50–179.50)	.006
Potassium (mmol/L)		4.27 (3.92–4.70)	4.25 (3.93–4.70)	4.40 (3.90–4.93)	<.001
Sodium (mmol/L)		138.67 (135.75–141.25)	139.00 (136.00–141.00)	138.33 (135.00–141.50)	.444
INR		1.33 (1.20–1.65)	1.30 (1.20–1.60)	1.40 (1.20–1.90)	<.001
PTT (s)		34.40 (29.10–44.63)	33.80 (28.90–43.55)	36.60 (29.80–51.30)	<.001
Creatinine (mg/dL)		1.25 (0.90–1.97)	1.20 (0.85–1.80)	1.67 (1.10–2.63)	<.001
BUN (mg/dL)		28.00 (18.57–44.67)	26.00 (18.00–41.00)	38.50 (25.67–58.00)	<.001
Comorbidities					
HTN, n (%)	No	2455 (77.52)	1973 (75.65)	482 (86.23)	<.001
	Yes	712 (22.48)	635 (24.35)	77 (13.77)	
AKI, n (%)	No	1599 (50.49)	1428 (54.75)	171 (30.59)	<.001
	Yes	1568 (49.51)	1180 (45.25)	388 (69.41)	
LC, n (%)	No	3021 (95.39)	2502 (95.94)	519 (92.84)	.002
	Yes	146 (4.61)	106 (4.06)	40 (7.16)	
CVA, n (%)	No	2806 (88.60)	2309 (88.54)	497 (88.91)	.801
	Yes	361 (11.40)	299 (11.46)	62 (11.09)	
CKD, n (%)	No	2018 (63.72)	1702 (65.26)	316 (56.53)	<.001
	Yes	1149 (36.28)	906 (34.74)	243 (43.47)	
CA, n (%)	No	2553 (80.61)	2128 (81.60)	425 (76.03)	.003
	Yes	614 (19.39)	480 (18.40)	134 (23.97)	
HLD, n (%)	No	1655 (52.26)	1357 (52.03)	298 (53.31)	.583
	Yes	1512 (47.74)	1251 (47.97)	261 (46.69)	
MI, n (%)	No	2721 (85.92)	2269 (87)	452 (80.86)	<.001
	Yes	446 (14.08)	339 (13)	107 (19.14)	
IHD, n (%)	No	1257 (39.69)	1041 (39.92)	216 (38.64)	.576
	Yes	1910 (60.31)	1567 (60.08)	343 (61.36)	
Sepsis, n (%)	No	1275 (40.26)	1175 (45.05)	100 (17.89)	<.001
	Yes	1892 (59.74)	1433 (54.95)	459 (82.11)	
Treatment					
Mechanical ventilation, n (%)	No	601 (18.98)	484 (18.56)	117 (20.93)	.194
	Yes	2566 (81.02)	2124 (81.44)	442 (79.07)	
Glucocorticoid use, n (%)	No	2882 (91)	2387 (91.53)	495 (88.55)	.026
	Yes	285 (9)	221 (8.47)	64 (11.45)	
Vasopressor use, n (%)	No	2552 (80.58)	2147 (82.32)	405 (72.45)	<.001
	Yes	615 (19.42)	461 (17.68)	154 (27.55)	
Antibiotic use, n (%)	No	2088 (65.93)	1792 (68.71)	296 (52.95)	<.001
	Yes	1079 (34.07)	816 (31.29)	263 (47.05)	

AG = anion gap, AKI = acute kidney injury, BUN = blood urea nitrogen, CA = cancer, CKD = chronic kidney disease, CVA = cerebrovascular accident, DBP = diastolic blood pressure, GCS = Glasgow Coma Scale, Hb = hemoglobin, HLD = hyperlipidemia, HR = heart rate, HTN = hypertension, IHD = ischemic heart disease, INR = international normalized ratio, LC = liver cirrhosis, MI = myocardial infarction, PLT = platelet, PTT = partial thromboplastin time, RBC = red blood cell, RDW = red blood cell distribution width, RR = respiratory rate, SAPS II = Simplified Acute Physiology Score II, SBP = systolic blood pressure, SOFA = Sequential Organ Failure Assessment, SPO_2_ = oxygen saturation, WBC = white blood cell.

### 3.2. Feature selection

To build the prediction model, LASSO regression and the Boruta algorithm were used to screen baseline features (Fig. 2). LASSO regression, based on 10-fold cross-validation at λ.min, selected variables with nonzero coefficients, including age, SOFA, SAPS II, GCS, acute kidney injury, hypertension, sepsis, HR, RR, red cell distribution width, AG, calcium, chloride, INR, partial thromboplastin time, and vasopressor use. Meanwhile, the Boruta algorithm, by comparing importance with randomly generated “shadow features,” selected the top 20 important feature variables, including SAPS II, SOFA, blood urea nitrogen, AG, serum creatinine, GCS, RR, sepsis, HR, acute kidney injury, age, chloride, oxygen saturation, temperature, vasopressor use, calcium, diastolic blood pressure, glucose, INR, and sodium. Finally, the intersection of features confirmed by both methods was determined as the predictors for model construction, totaling 14 variables: age, SOFA, SAPS II, GCS, acute kidney injury, sepsis, HR, RR, oxygen saturation, AG, calcium, chloride, INR, and vasopressor use.

**Figure 2.**
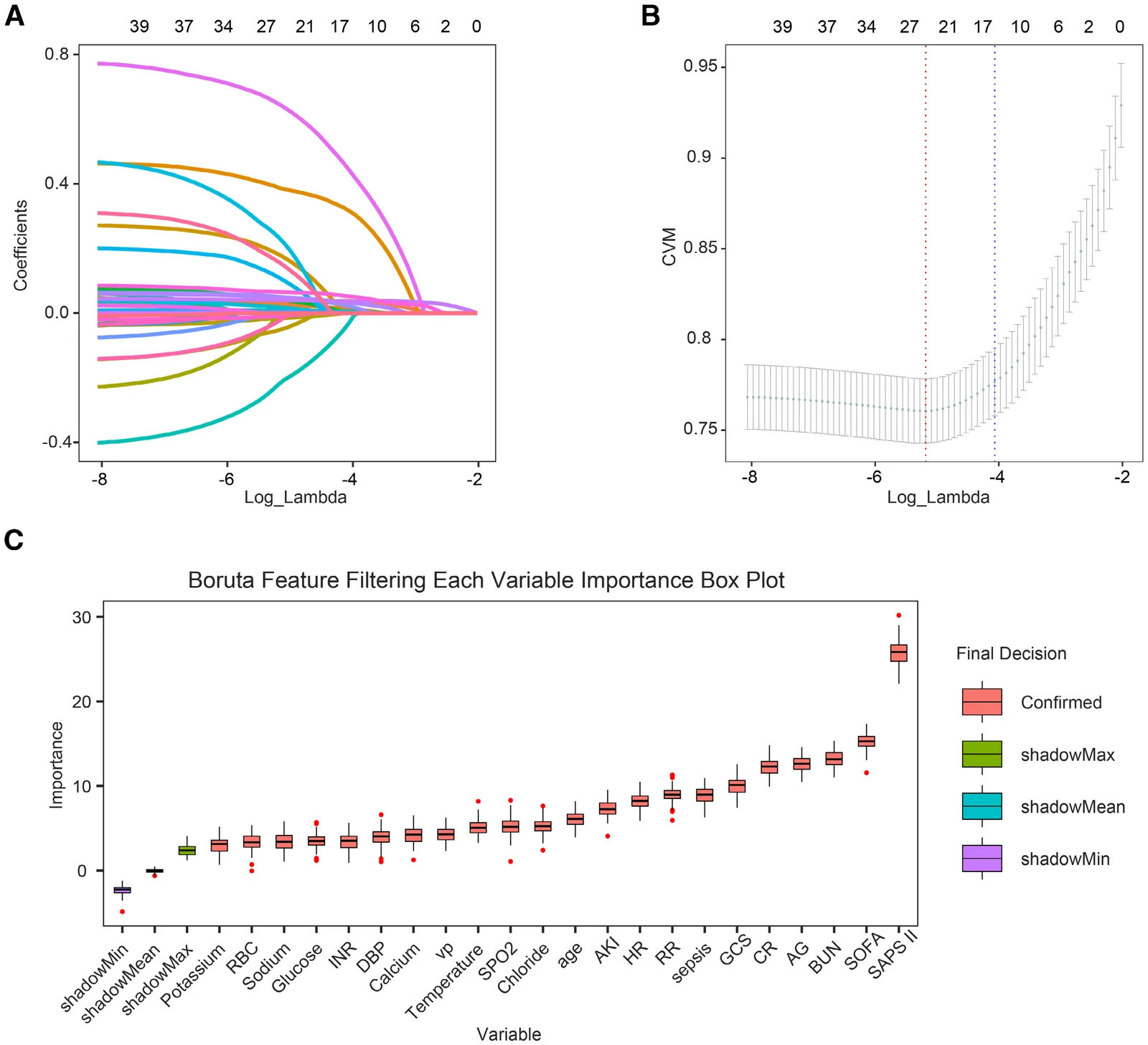
Feature selection using LASSO regression and the Boruta algorithm. (A) LASSO regression lambda and coefficients plot. (B) The relationship between lambda (regularization parameter) and CVM (mean cross-validation error) in LASSO regression. (C) Boruta algorithm feature importance ranking. AG = anion gap, AKI = acute kidney injury, BUN = blood urea nitrogen, CR = creatinine, DBP = diastolic blood pressure, GCS = Glasgow Coma Scale, HR = heart rate, INR = international normalized ratio, LASSO = least absolute shrinkage and selection operator, RBC = red blood cell, RR = respiratory rate, SAPS II = Simplified Acute Physiology Score II, SOFA = Sequential Organ Failure Assessment, SPO_2_ = oxygen saturation.

### 3.3. Model development and comparison

Based on the 14 selected features, this study compared the predictive performance of 5 machine learning models on the internal validation set (Fig. 3, Table 2). All models demonstrated certain predictive capability. Among them, SVM had the highest AUROC (0.789), followed closely by CatBoost (0.788). In terms of overall performance, the CatBoost model had the highest F1 score (0.498), showing relatively balanced performance; the Light Gradient Boosting Machine model had the highest accuracy (0.841) and specificity (0.935), indicating its strong ability to identify surviving patients. The SVM model showed the highest sensitivity (0.675), indicating better performance in identifying non-survivors. The calibration curve showed that the CatBoost model demonstrated good calibration compared with the other models, with its predicted probabilities showing reasonable agreement with observed outcomes across most risk strata (Brier score: 0.120). DCA indicated that within most clinically meaningful decision threshold ranges, the CatBoost model provided relatively stable clinical net benefit. Sensitivity analyses showed that the CatBoost model’s AUROC remained stable across different imputation methods (Supplementary Table 1, Supplemental Digital Content 1). Comprehensively considering these factors, the CatBoost model was selected as the optimal model for subsequent external validation and interpretability analysis.

**Table 2:** Performance comparison of different machine learning models in internal validation.

Model	AUROC	F1 score	Sensitivity	Specificity	Accuracy	Brier score
CatBoost	0.788	0.498	0.482	0.900	0.825	0.120
SVM	0.789	0.498	0.675	0.773	0.756	0.119
LGBM	0.764	0.482	0.412	0.935	0.841	0.123
RF	0.762	0.494	0.535	0.862	0.803	0.124
XGB	0.738	0.404	0.342	0.923	0.819	0.130

AUROC = area under the receiver operating characteristic curve, CatBoost = Categorical Boosting, LGBM = Light Gradient Boosting Machine, RF = Random Forest, SVM = Support Vector Machine, XGB = eXtreme Gradient Boosting.

**Figure 3.**
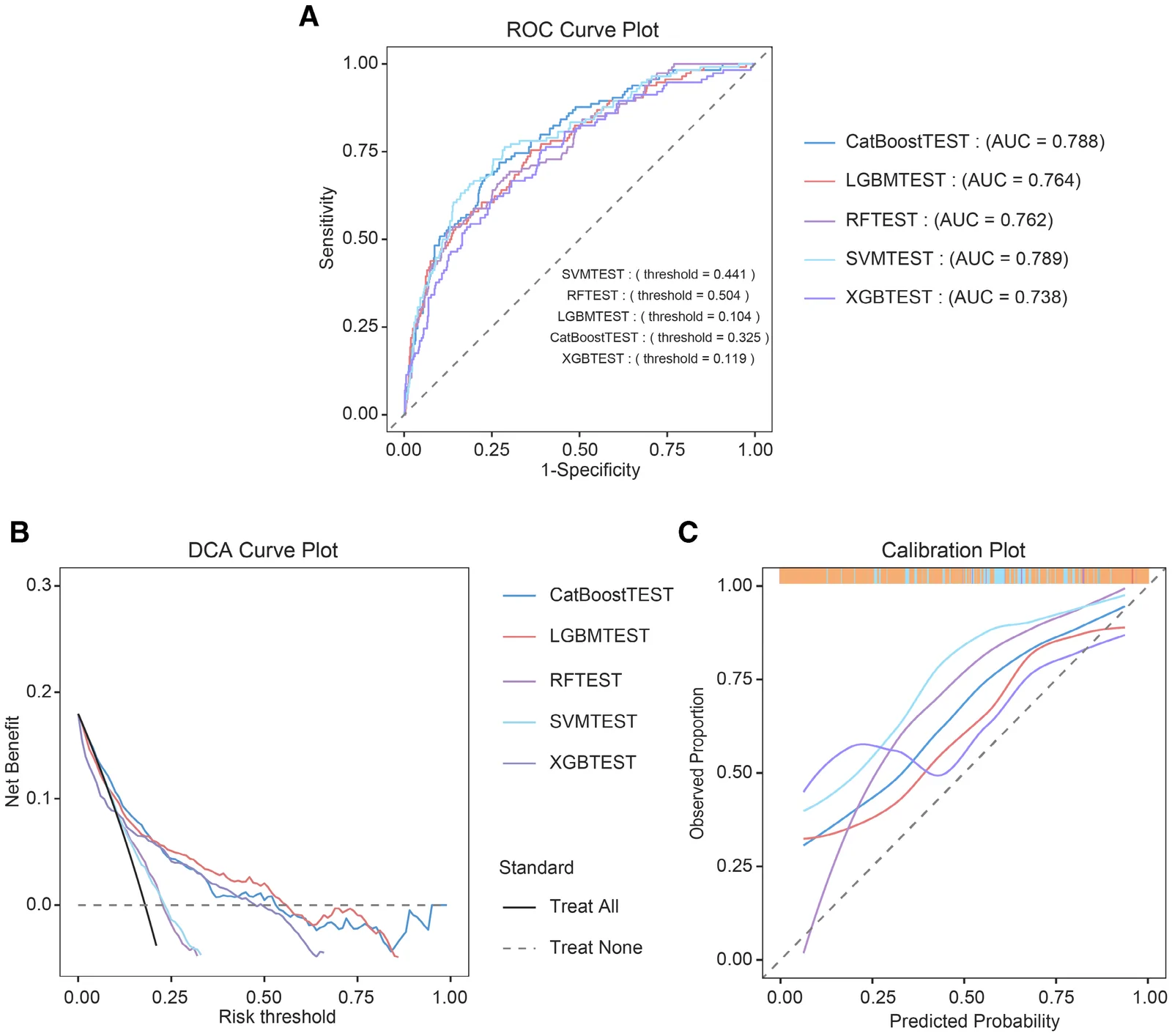
Performance comparison of 5 machine learning models in internal validation. (A) Receiver operating characteristic curves. (B) Decision curve analysis. (C) Calibration curves. AUC = area under the curve, CatBoost = Categorical Boosting, DCA = decision curve analysis, LGBM = Light Gradient Boosting Machine, RF = Random Forest, ROC = receiver operating characteristic, SVM = Support Vector Machine, XGB = eXtreme Gradient Boosting.

### 3.4. External validation

To evaluate the model’s generalizability, the best-performing CatBoost model from MIMIC-IV was externally validated on the independent eICU-CRD dataset (n = 1781). The results showed that the model achieved an AUROC of 0.739 in this external cohort (Fig. 4), which was slightly lower than that in the internal validation but still maintained reasonable discriminatory ability. The results suggest that the model has potential for generalization across ICU patient populations from different sources.

**Figure 4.**
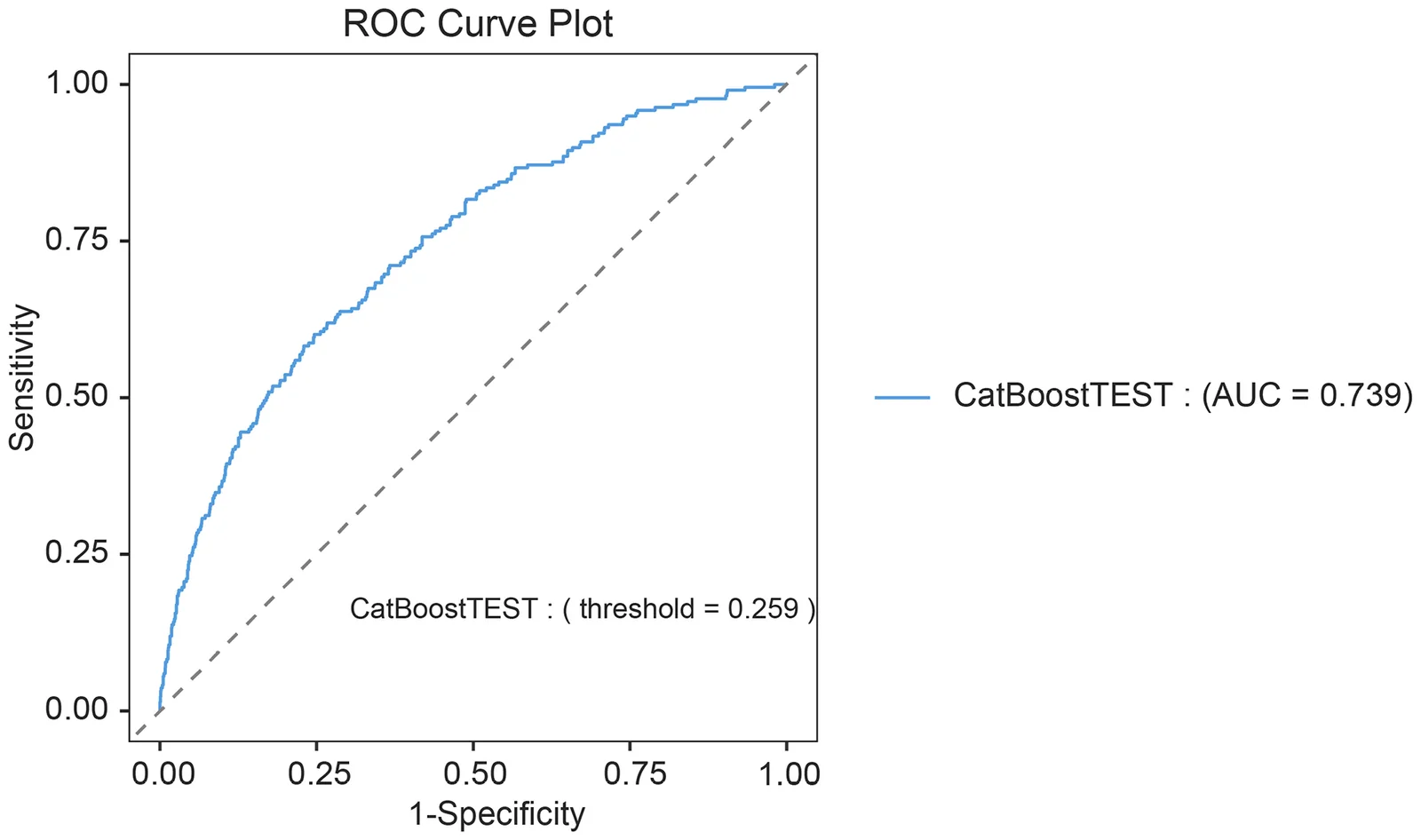
External validation of the CatBoost model in the eICU-CRD cohort (ROC curve). AUC = area under the curve, CatBoost = Categorical Boosting, eICU-CRD = eICU Collaborative Research Database, ROC = receiver operating characteristic.

### 3.5. Model interpretability analysis

To further quantify the contribution of each feature to predicting the 28-day mortality risk in ICU patients with COPD complicated by HF, the SHAP method was used for interpretability analysis of the optimal CatBoost model (Fig. 5). The analysis showed that disease severity scores (SOFA, SAPS II), AG, vasopressor use, and RR were the top 5 most important features influencing the model’s predictions. Other key features included age, HR, GCS, INR, sepsis, and some electrolyte indicators. The bees plot further revealed the association patterns between these features and predicted risk. The above analysis results highlight the important role of organ function scores, key vital signs, and clinical interventions in the model’s risk assessment.

**Figure 5.**
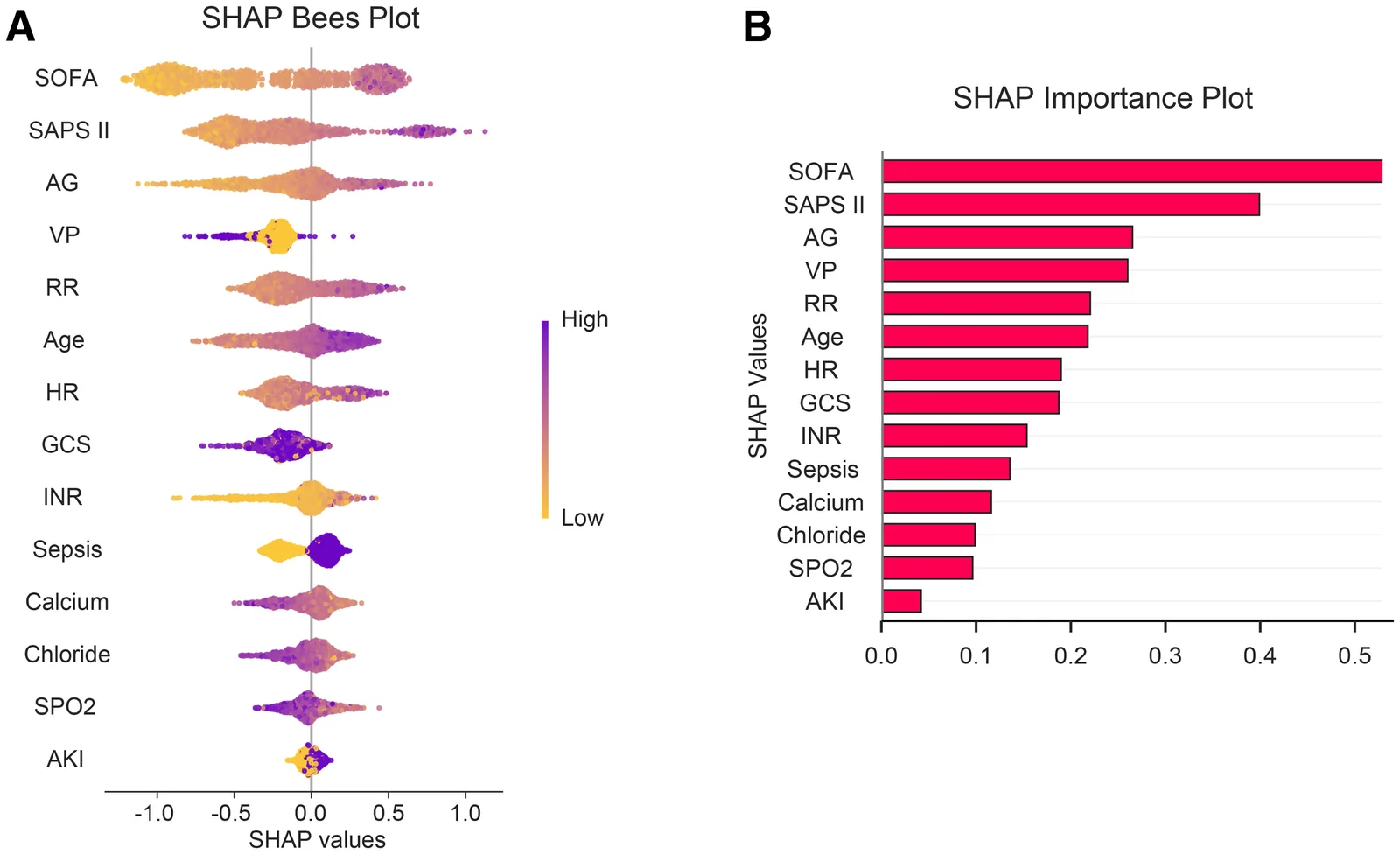
SHAP interpretability analysis of the CatBoost model. (A) SHAP bees plot. (B) SHAP importance plot. AG = anion gap, AKI = acute kidney injury, CatBoost = Categorical Boosting, GCS = Glasgow Coma Scale, HR = heart rate, INR = international normalized ratio, SHAP = Shapley Additive Explanations, SAPS II = Simplified Acute Physiology Score II, SOFA = Sequential Organ Failure Assessment, SPO_2_ = oxygen saturation, VP = vasopressor.

## 4. Discussion

This study developed and externally validated multiple machine learning models for the early prediction of 28-day mortality risk in ICU patients with COPD complicated by HF. In internal validation, the AUROC difference between SVM (0.789) and CatBoost (0.788) was minimal among the 5 models. However, CatBoost demonstrated more balanced overall performance in calibration and DCA and was therefore selected as the optimal model. This model also showed good discrimination in external validation (AUROC 0.739). SHAP analysis identified SOFA score, SAPS II score, AG, vasopressor use, and RR as the most important predictive factors. These results indicate that machine learning models based on routine early admission clinical data can facilitate early risk identification in this high-risk comorbid population and support clinical decision-making in the ICU.

COPD and HF can mutually exacerbate each other. The bidirectional mechanism manifests as pulmonary congestion and low cardiac output due to HF worsening respiratory function, while COPD-related hypoxic pulmonary hypertension and systemic inflammation aggravate cardiac load and remodeling.^[5]^ Previous studies have confirmed that patients with combined COPD and HF have a higher mortality risk,^[15]^ and some attempts have been made to build prognostic prediction models for such patients.^[7,16]^ However, most previous models were based on single-center data, lacked rigorous external validation, and had limited clinical interpretability. To address these limitations, this study developed and validated an interpretable machine learning model using multicenter ICU data. It employed the LASSO-Boruta algorithm for feature selection, systematically compared 5 machine learning algorithms, and identified CatBoost as the optimal model (internal validation AUROC, 0.788; external validation AUROC, 0.739). Furthermore, this study provided a quantitative interpretation of the model’s decisions through the SHAP framework, identifying key risk drivers such as SOFA score, AG, vasopressor use, and RR, making its predictions easier to integrate into clinical practice.

This study used the SHAP framework for interpretability analysis of the prediction model to elucidate the contribution of various clinical features to mortality risk in ICU patients with COPD complicated by HF and the underlying pathophysiological connections. The analysis showed that organ function and disease severity scores (SOFA and SAPS II) are important predictors. The SOFA score is a widely used organ function assessment tool in critical care, and the SAPS II score excels in predicting ICU patient mortality risk. Both objectively reflect the patient’s physiological state and illness severity; their elevation typically indicates more severe physiological derangement and organ dysfunction, heralding a poor prognosis.^[17,18]^ Such comorbid patients often exhibit significantly increased RRs, suggesting insufficient respiratory compensation.^[19]^ Regarding laboratory indicators, reduced cardiac output from HF combined with hypoxemia from COPD leads to systemic tissue oxygen supply insufficiency, enhancing anaerobic metabolism and promoting lactate accumulation. Concurrently, systemic inflammation and frequently concomitant renal insufficiency in these patients further aggravate metabolic acidosis.^[20–22]^ Therefore, elevated AG not only indicates the severity of acidosis but also marks systemic metabolic imbalance, which is closely associated with adverse outcomes. On the other hand, INR is a key indicator for assessing the extrinsic coagulation pathway. In critically ill patients with combined COPD and HF, systemic inflammation, hypoxia, and endothelial injury collectively lead to significant coagulation dysfunction, manifesting as a hypercoagulable state.^[23]^ These patients are at higher risk of thrombosis and are more likely to receive anticoagulants, which can elevate INR.^[16]^ Additionally, the risk of sepsis, a core clinical syndrome centered on organ dysfunction, is significantly increased in this population. HF may elevate sepsis risk through inflammation pathways mediated by factors such as β-nerve growth factor, while the chronic systemic inflammation and prothrombotic state in COPD can further amplify the immune dysregulation and organ damage caused by sepsis, collectively worsening patient prognosis.^[24–26]^ At the treatment level, the use of vasoactive drugs directly reflects hemodynamic instability, typically indicating that the disease has entered a critical stage.^[7]^ In summary, SHAP analysis enhances the model’s clinical interpretability by quantifying feature contributions and revealing their pathophysiological links.

Several limitations of this study should be noted. First, the data originate from US ICU databases, and the characteristics of their patient populations may differ from those in other regions, limiting the model’s generalizability. Future external validation in broader, multi-geographic populations is needed. Second, the predictive variables incorporated into this model were all static data from the first 24 hours of ICU admission, failing to include dynamic changes during treatment or some important specific examination results (e.g., pulmonary function tests and echocardiography), potentially omitting some valuable prognostic predictors. Furthermore, although the model demonstrated reasonable discrimination, its performance still has room for further optimization. Additionally, due to computational resource constraints, we did not perform extensive hyperparameter tuning for the machine learning models, which may have prevented the models from achieving optimal performance. Finally, although SHAP analysis improves model interpretability, its results may be influenced by the model’s inherent biases, thus limiting its ability to fully capture true feature associations.^[27]^ Future work could involve deploying the model as a web-based tool or mobile application to facilitate real-time risk assessment. Clinicians would input patient data into a user-friendly interface, and the model would instantly return the predicted mortality risk along with SHAP-based explanations of key contributing factors. Such a platform could enable rapid risk stratification at the bedside and support clinical decision-making in the ICU.

## 5. Conclusion

This study developed and validated an interpretable machine learning model for the early prediction of 28-day mortality risk in ICU patients with COPD complicated by HF, based on large-scale multicenter ICU data. By combining the LASSO-Boruta algorithm for robust feature selection and systematically comparing multiple machine learning algorithms, the CatBoost model was ultimately selected as the optimal prediction tool. The model demonstrated stable predictive performance in both internal and external validation (AUROCs of 0.788 and 0.739, respectively). Additionally, this study applied the SHAP framework to provide a quantitative interpretation of the model’s decision-making process, identifying core predictors including SOFA score, SAPS II score, AG, vasopressor use, and RR. These factors are closely linked to the underlying pathophysiology, thereby improving the model’s clinical interpretability.

## Acknowledgments

The authors gratefully acknowledge all contributors to this research.

## Author contributions

**Conceptualization:** Jiayi Shen, Yucheng Liu, Yikun Chen.

**Data curation:** Jiayi Shen, Yucheng Liu, Yikun Chen.

**Formal analysis:** Jiayi Shen, Yucheng Liu.

**Methodology:** Jiayi Shen.

**Project administration:** Yikun Chen.

**Supervision:** Yikun Chen.

**Visualization:** Jiayi Shen.

**Writing – original draft:** Jiayi Shen.

**Writing – review & editing:** Yucheng Liu, Yikun Chen.


