# Supplementary material for: Development and validation of an interpretable machine learning model for early prediction of mortality in ICU patients with chronic obstructive pulmonary disease and heart failure
Source: Medicine (Baltimore). 2026 Oct 23;105(43):e51224. doi: 10.1097/MD.0000000000051224 (PMC13706389; doi:10.1097/MD.0000000000051224)
Supplement: Supplementary file 1 [file medi-105-e51224-s001.docx]

| **Supplementary Table 1 Performance of CatBoost under different missing data imputation methods** | | | | | |
| --- | --- | --- | --- | --- | --- |
| **Imputation method** | **AUROC** | **F1-Score** | **Sensitivity** | **Specificity** | **Accuracy** |
| KNN (primary analysis) | 0.788 | 0.498 | 0.482 | 0.900 | 0.825 |
| Mean imputation | 0.788 | 0.509 | 0.500 | 0.898 | 0.826 |
| Complete-case analysis | 0.787 | 0.472 | 0.511 | 0.842 | 0.778 |
